# Serum lipid mediator profiles in COVID-19 patients and lung disease severity: a pilot study

**DOI:** 10.1038/s41598-023-33682-2

**Published:** 2023-04-20

**Authors:** Pilar Irún, Rafael Gracia, Elena Piazuelo, Julián Pardo, Elena Morte, José Ramon Paño, Julio Boza, Patricia Carrera-Lasfuentes, Gustavo A. Higuera, Angel Lanas

**Affiliations:** 1 Centro de Investigación Biomédica en Red de Enfermedades Hepáticas y Digestivas (CIBEREHD), Instituto de Salud Carlos III (ISCIII), Zaragoza, Spain; 2 Instituto de Investigación Sanitaria Aragón (IIS Aragón), Zaragoza, Spain; 3 SOLUTEX GC, SL, Zaragoza, Spain; 4 Instituto Aragonés de Ciencias de la Salud (IACS Aragón), Zaragoza, Spain; 5 Departamento de Farmacología y Fisiología, Facultad de Medicina, Universidad de Zaragoza, Zaragoza, Spain; 6 Departamento de Microbiología, Medicina Preventiva y Salud, Universidad de Zaragoza, Zaragoza, Spain; 7 Aragón I + D Foundation (ARAID), Government of Aragon, Zaragoza, Spain; 8 Centro de Investigación Biomédica en Red de Enfermedades Infecciosas, Instituto de Salud Carlos III (ISCIII), Zaragoza, Spain; 9 Infectious Disease Department, University Hospital Lozano Blesa, Zaragoza, Spain; 10 Service of Digestive Diseases, Hospital Clínico Universitario Lozano Blesa, Zaragoza, Spain; 11 Departamento de Medicina, Psiquiatría y Dermatología, Facultad de Medicina, Universidad de Zaragoza, Zaragoza, Spain

**Keywords:** Viral infection, Lipidomics

## Abstract

Coronavirus disease 2019 (COVID-19) caused by SARS-CoV-2 infection is highly heterogeneous, ranging from asymptomatic to severe and fatal cases. COVID-19 has been characterized by an increase of serum pro-inflammatory cytokine levels which seems to be associated with fatal cases. By contrast, the role of pro-resolving lipid mediators (SPMs), involved in the attenuation of inflammatory responses, has been scarcely investigated, so further studies are needed to understand SPMs metabolism in COVID-19 and other infectious diseases. Our aim was to analyse the lipid mediator metabolome, quantifying pro- and anti-inflammatory serum bioactive lipids by LC–MS/MS in 7 non-infected subjects and 24 COVID-19 patients divided into mild, moderate, and severe groups according to the pulmonary involvement, to better understand the disease outcome and the severity of the pulmonary manifestations. Statistical analysis was performed with the R programming language (R Foundation for Statistical Computing, Vienna, Austria). All COVID-19 patients had increased levels of Prostaglandin E_2_. Severe patients showed a significant increase versus controls, mild- and moderate-affected patients, expressed as median (interquartile range), in resolvin E1 [112.6 (502.7) vs 0.0 (0.0) pg/ml in the other groups], as well as in maresin 2 [14.5 (7.0) vs 8.1 (4.2), 5.5 (4.3), and 3.0 (4.0) pg/ml, respectively]. Moreover, 14-hydroxy docosahexaenoic acid (14-HDHA) levels were also increased in severe vs control and mild-affected patients [24.7 (38.2) vs 2.4 (2.2) and 3.7 (6.4) ng/mL, respectively]. Resolvin D5 was also significantly elevated in both moderate [15.0 (22.4) pg/ml] and severe patients [24.0 (24.1) pg/ml] versus controls [0.0 (0.0) pg/ml]. These results were confirmed by sparse partial least squares discriminant analysis which highlighted the contribution of these mediators to the separation between each of the groups. In conclusion, the potent inflammatory response to SARS-CoV-2 infection involves not only pro- but also anti-inflammatory lipid mediators that can be quantified in easily accessible serum samples, suggesting the need to perform future research on their generation pathways that will help us to discover new therapeutic targets.

## Introduction

Infection with severe acute respiratory syndrome coronavirus-2 (SARS-CoV-2) is the cause of the pandemic coronavirus disease 2019 (COVID-19) which shows substantial phenotypic variability, ranging from an asymptomatic to a severe life-threatening disease. The occurrence of acute respiratory distress syndrome (ARDS), which needs intubation in intensive care units (ICUs), is the key feature of COVID-19 severity^1^. Apart from that, the presence of other underlying, chronic diseases and comorbidities compromising organ functioning, such as cardiovascular disease, liver disease, cancer, and diabetes, among others, increases the risk of death in these patients^2–4^. Most severe COVID-19 symptoms are connected with the deregulation of inflammatory processes. The inflammatory response is a key protective immune system mechanism to counteract harmful stimuli such as an infection^5,6^ that must be well-balanced and properly resolved to reach tissue homeostasis after pathogen clearance. Many studies have confirmed that severe COVID-19 patients suffer from a hyperinflammatory response that resembles the cytokine storm observed in sepsis, characterized by an uncontrolled release of pro-inflammatory cytokines that would trigger the different mechanisms responsible for tissue damage, including coagulation disorders, endothelial damage, and lung disease, during COVID-19 and it has been shown to correlate with disease severity and mortality^7–9^. A recently published article describes how unresolved focal airway inflammation results in further lung tissue damage in non-survival intubated COVID-19 patients: starting with increased pro-inflammatory mediator production; which activates lung macrophages and neutrophils; followed by reactive oxygen species release; that leads to increased peroxidation and overexpression of matrix metalloproteinases (MPPs), mainly MMP-2 and MMP-8^10^. This hyperinflammatory state worsened by the cytokine storm contains a unique lipid profile signature. An immune-mediated inflammatory hypolipidemia is caused, that is characterized by reduced levels of low- and high-density lipoprotein cholesterol (LDL-C, HDL-C), total cholesterol and apolipoproteins E and A1; and also by increased levels of triglycerides (TG) and lipoprotein oxidation^11–13^. This lipid imbalance can be explained by SARS-CoV-2 virus need to hijack the host’s metabolic machinery and lipid resources for its own replication. The early phase of the inflammatory mechanism also includes the release of polyunsaturated fatty acids (PUFAs) from the membrane of some cells for their conversion to pro-inflammatory lipid mediators or eicosanoids. This includes prostaglandins (PGs), leukotrienes (LTs), and tromboxanes (TXs) that activate the innate immune response. At this stage, LTB_4_ acts as a chemotactic metabolite for neutrophil recruitment and influx to the infected sites. Also, when PGE_2_ and PGD_2_ are switched on, these lipid mediators promote a shift to production of anti-inflammatory and specialized pro-resolving lipid mediators (SPMs). SPMs block neutrophil recruitment, thereby: regulating cytokine and chemokine production and skewing macrophages from M1 to M2 type. As a result, SPMs enhance macrophage phagocytosis of apoptotic polymorphonuclear leukocytes, cellular debris, and pathogen killing^14^. SPMs, namely RvD1 and MaR1 have been probed to control inflammation and tissue degradation through reduction of MMPs activity. Specifically, RvD1 was administered to a mice model of skin disease caused by UVB irradiation^15^. Furthermore, MaR1 was tested in a rat model of vascular cognitive impairment caused by chronic cerebral hypoperfusion^16^. In this sense, previous studies have shown that SARS-CoV-2 infection is associated with high production of fatty-acid-derived lipid mediators, which is directly correlated with the severity of COVID-19^17,18^. However, little is known about the contribution of SPMs to COVID-19. Most SPMs, such as resolvins (Rv), protectins (PD), and maresins (MaR), are synthesized from ω3-polyunsaturated fatty acids (PUFAs), and lipoxins (LX) from arachidonic acid (AA). SPMs are drivers of the inflammation resolution phase and critical for activating the mechanism involved in tissue repair and homeostasis^19^. Finally, lipoprotein dysregulation and SPMs release has also been strongly linked in other diseases, such as sepsis, and operating via the above-mentioned processes^20^.

Previous studies in patients with acute lung injury/ARDS and severe sepsis/septic shock needing mechanical ventilation, something common in severe COVID-19, have revealed that ω3-PUFA supplementation reduces mortality, organ failure, and ICU times, and improves oxygenation and clinical outcomes^21–24^. Research on animal models of inflammation has also shown beneficial effects of fish oil, rich in ω3-PUFAs, concerning pulmonary microvascular protein permeability and reduction of pulmonary neutrophil accumulation^25,26^. For these reasons, the use of parenteral ω3-PUFA supplementation has been suggested as an alternative to treat patients suffering from severe COVID-19^27,28^; finally, a randomized study (NCT04335032) is currently ongoing to investigate its effects in hospitalized subjects with confirmed SARS-CoV-2.

From the clinical perspective of hospital admissions rates due to lung complications caused by SARS-CoV-2 infection and the potential role of SPMs in resolving inflammation and tissue repair, a detailed characterization of the possible alterations in SPM profile after infection would be useful to classify patients according to disease prognosis and is crucial to determine the suitability of future treatments with ω-3 PUFAs or SPMs that have been proposed. Our aim was to perform the lipidomic profiling of hospitalized patients suffering from COVID-19 classified according to the oxygen therapy needs.

## Results

### Demographics

**Figure 1:**
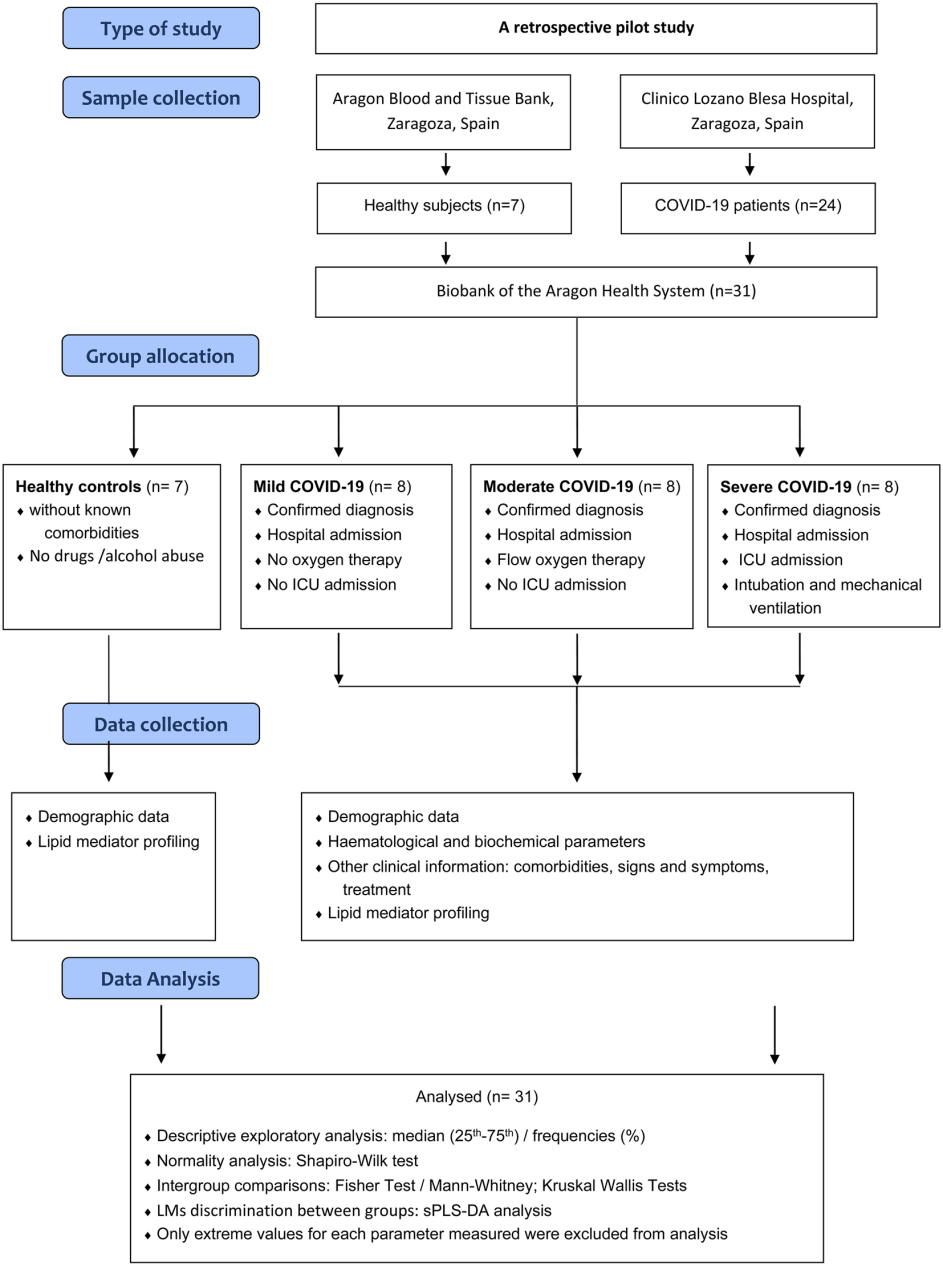
Study flow diagram. Schematic representation summarizing the type of study, sample collection, group allocation, data collection, and analysis.

This is a retrospective pilot study including samples from 31 participants: 24 COVID-19 patients that were hospitalized in the Clinico Lozano Blesa Hospital in Zaragoza, Spain, in the first wave of the pandemic and seven healthy subjects collected among voluntary blood donors recruited in the Aragon Blood and Tissue Bank. COVID-19 patients were allocated into three groups (n = 8) according to the severity criteria at the time of hospital admission, depending on oxygen therapy needs: mild cases that do not need oxygen therapy, moderate cases needing flow oxygen therapy, and severe cases with severe ARDS that requires intubation and mechanical ventilation in ICUs (Fig. 1).

No significant differences were observed between COVID-19 groups with different disease severity or between those and the control group regarding gender distribution or time between the onset of symptoms and blood withdrawal (COVID-19 groups). Age distribution only showed significant differences (Table 1) between the control group and both the moderate and severe COVID-19 groups (*P* = 0.013 and 0.015, respectively). The most common reported symptoms of the disease (Table 2) were fever (83%), cough (63%), dyspnoea (46%), and diarrhoea (25%), followed by sputum production and muscle pain or fatigue (13%). Finally, dysgeusia (8%) and other symptoms like skin exanthem, headache, odynophagia, haemoptysis, sickness, or asthenia were reported only in one of 24 patients (4%). Viral pneumonia caused by SARS-CoV-2 was diagnosed in 67% of cases. The mean ± SD time from symptom onset to blood extraction was 6.6 ± 3.8 days. There were no significant differences between the COVID-19 groups in the blood levels of alanine or aspartate aminotransferases, C-reactive protein, creatinine kinase, creatinine, chloride, sodium, potassium, basophils, erythrocytes, lymphocyte and platelet counts, prothrombin time, nor in cardiac troponin T. When comparing different COVID-19 groups, the levels of two parameters, activated partial thromboplastin time (aPTT, in the severe group) and blood urea nitrogen (BUN, in the mild group), were significantly lower than those of the other two COVID-19 groups (Table 1, *P* < 0.05 in all two-group comparisons). Haematological and biochemical variables are shown in Table 1. As expected, there were more deaths (Table 2) in the severe versus mild COVID-19 group of patients (*P* = 0.007).

**Table 1:** Patient demographics and haematological and biochemical variables.

	Control (n = 7)	Mild (n = 8)	Moderate (n = 8)	Severe (n = 8)	*P* -value
Demographics					
Age (years)	45.0 (43.5–45.5)	50.5 (40.8–78.5)	79.0 (65.3–87.0)	69.0 (60.3–82.3)	**0.015**^**a**^ **0.013**^**b**^
Gender					0.606
Male	6 (85.7%)	5 (62.5%)	4 (50.0%)	6 (75.0%)	
Female	1 (14.3%)	3 (37.5%)	4 (50.0%)	2 (25.0%)	
Blood determinations					
ALT, μkat/l		0.28 (0.21–0.56)	0.40 (0.20–0.65)	0.62 (0.53–1.11)	0.082
AST, μkat/l		0.40 (0.34–0.56)	0.58 (0.38–1.18)	0.78 (0.63–1.01)	0.057
CRP, mg/l		52.6 (8.9–104.3)	69.8 (51.1–142.9)	131.8 (48.5–201.0)	0.531
Chloride, mmol/l		101.3 (98.4–102.5)	103.1 (100.1–111.3)	101.0 (96.1–102.7)	0.267
CK, μkat/l		1.03 (0.70–1.42)	3.23 (1.26–8.34)	1.68 (0.86–1.95)	0.224
aPTT, s		32.3 (30.1–33.7)	31.8 (31.2–34.0)	28.0 (26.6–29.9)	**0.021**^**a**^ **0.010**^**c**^
Creatinine, μmol/l		83.5 (67.9–98.4)	83.1 (77.8–100.8)	111.4 (77.8–192.9)	0.383
Basophils count, 10^9^/l		0.02 (0.01–0.03)	0.01 (0.01–0.02)	0.01 (0.01–0.04)	0.315
Eosinophils count, 10^9^/l		0.09 (0.01–0.13)	0.00 (0.00–0.01)	0.00 (0.00–0.01)	**0.034**^**a**^ **0.035**^**b**^
Erythrocytes count, 10^12^/l		4.3 (4.1–4.8)	4.2 (3.8–5.3)	4.1 (4.0–4.1)	0.357
Hematocrit, l/l		0.40 (0.37–0.45)	0.37 (0.36–0.46)	0.36 (0.35–0.38)	0.103
Lymphocytes count, 10^9^/l		0.83 (0.52–1.18)	0.74 (0.63–0.98)	0.51 (0.44–0.61)	0.229
Platelets count, 10^9^/l		167.5 (141.8–217.8)	177.0 (149.0–193.5)	225.0 (144.3–254.3)	0.481
Potassium, mmol/l		4.0 (3.8–4.1)	3.9 (3.8–4.0)	3.8 (3.3–4.5)	0.918
PT, s		15.5 (13.2–17.8)	13.8 (13.3–17.0)	14.2 (13.5–15.5)	0.822
Sodium, mmol/l		139.5 (137.0–141.0)	141.5 (139.3–150.3)	138.0 (134.0–140.0)	0.172
Cardiac troponin T, μg/l		0.01 (0.01–0.02)	0.01 (0.01–0.02)	0.02 (0.01–0.04)	0.528
BUN, mmol/l		8.7 (7.9–13.1)	17.1 (14.3–18.7)	24.8 (22.1–48.2)	**0.027**^**a**^ **0.049**^**b**^

Patients’ data were compared using Fisher’s exact test for categorical variables. Kruskal–Wallis test was used for continuous variables, if *p* < 0.05, the U de Mann–Whitney test was applied. Significant differences are reported in these cases. Continuous variables are given as medians and interquartile ranges (25th–75th percentile) and categorical ones by the number of patients and percentage, n (%). The Mann–Whitney *U* test was applied between (^a^) severe and mild, (^b^) moderate and mild, and (^c^) severe and moderate groups. ALT, alanine aminotransferase; Abbreviations: aPTT, activated partial thromboplastin time; AST, aspartate aminotransferase; BUN, blood urea nitrogen; CK, creatinine kinase; CPR, C-reactive protein; PT, prothrombin time.

Significant values are in [bold].

**Table 2:** Clinical information for COVID-19 patients.

	Mild (n = 8)	Moderate (n = 8)	Severe (n = 8)	*P* -value
Comorbidities				
Diabetes	1 (12.5%)	2 (25.0%)	1 (12.5%)	1.000
Hypertension	3 (37.5%)	3 (37.5%)	6 (75.5%)	0.386
Cardiovascular disease	3 (37.5%)	1 (12.5%)	2 (25.0%)	0.837
Chronic obstructive pulmonary disease	0 (0.0%)	2 (25.0%)	1 (12.5%)	0.747
Malignancy	1 (12.5%)	0 (0.0%)	2 (25.0%)	0.747
Chronic renal disease	3 (37.5%)	1 (12.5%)	1 (12.5%)	0.577
Dyslipidemia	3 (37.5%)	2 (25.0%)	3 (37.5%)	1.000
Stroke (ACV)	0 (0.0%)	2 (25.0%)	0 (0.0%)	0.304
Signs and symptoms				
Fever	8 (100.0%)	6 (75.0%)	6 (75.5%)	0.494
Cough	6 (75.5%)	5 (62.5%)	4 (50.0%)	0.866
Sputum production	3 (37.5%)	0 (0.0%)	0 (0.0%)	0.083
Skin exanthem	1 (12.5%)	0 (0.0%)	0 (0.0%)	1.000
Dyspnoea	5 (62.5%)	3 (37.5%)	3 (37.5%)	0.670
Pneumonia	4 (50.0%)	5 (62.5%)	7 (87.5%)	0.163
Diarrhoea	2 (25.0%)	2 (25.0%)	2 (25.0%)	1.000
Myalgia or fatigue	3 (37.5%)	0 (0.0%)	0 (0.0%)	0.083
Headache	1 (12.5%)	0 (0.0%)	0 (0.0%)	1.000
Odynophagia	0 (0.0%)	0 (0.0%)	1 (12.5%)	1.000
Hemoptysis	1 (12.5%)	0 (0.0%)	0 (0.0%)	1.000
Dysgeusia	0 (0.0%)	1 (12.5%)	1 (12.5%)	1.000
Tachypnea	0 (0.0%)	1 (12.5%)	0 (0.0%)	1.000
Asthenia	0 (0.0%)	0 (0.0%)	1 (12.5%)	1.000
Treatment^a^				
Antiviral therapy (lopinavir/ritonavir, remdesivir)	2 (33.3%)	3 (37.5%)	6 (100.0%)	**0.030**^**b**^ **0.028**^**c**^
Antibiotic therapy (ceftriaxone, azithromycin)	1 (16.7%)	6 (75.0%)	2 (33.3%)	0.136
Anticoagulants (bemiparin, enoxaparin)	1 (16.7%)	3 (37.5%)	1 (16.7%)	0.675
Immunomodulators	5 (83.3%)	3 (37.5%)	5 (83.3%)	0.179
Anti-cytokines (Tocillizumab)	0 (0.0%)	0 (0.0%)	1 (16.7%)	0.600
Corticosteroids (dexamethasone, prednisone)	3 (50.0%)	3 (37.5%)	3 (50.0%)	1.000
Hydroxychloroquine	3 (50.0%)	3 (37.5%)	3 (50.0%)	1.000
Interferons	0 (0.0%)	0 (0.0%)	1 (16.7%)	0.600
Exitus, n (%)	0 (0.0%)	3 (37.5%)	6 (75.0%)	**0.003**^**b**^

Patients’ data were compared using Fisher’s exact test. Categorical variables are given as number and percentage of patients, n (%). (^a^) Information was missing for 2 mild and 2 severe patients. Percentages in these cases are calculated for n = 6. Fisher’s exact test was applied (^b^) between mild and severe COVID-19 groups and (^c^) between moderate and severe COVID-19 groups (no significant differences between the other groups). The absence of superscript numbers in *P*-values indicates that comparisons were made between the three groups.

Significant values are in [bold].

### Lipid mediator metabolome analysis

**Figure 2:**
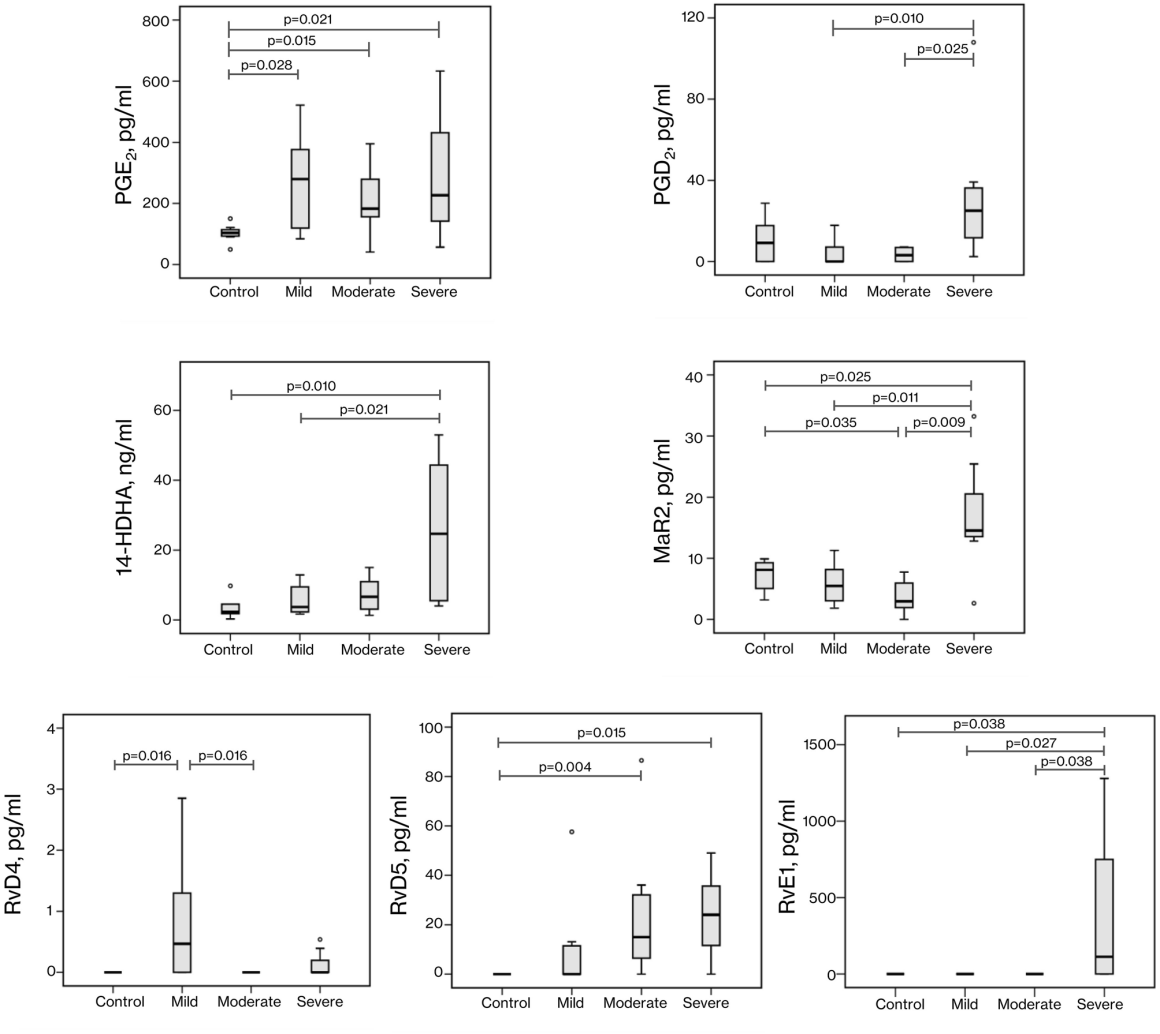
Serum levels of active lipid mediators and ω3-PUFAs differentially expressed in healthy controls or COVID-19 patients with different severity. COVID-19 patients were divided into three groups: mild, moderate, and severe pulmonary involvement. Panels represent concentrations of prostaglandin E_2_ (PGE_2_), prostaglandin D_2_ (PGD_2_), 14-hydroxy docosahexaenoic acid (14-HDHA), maresin 2 (MaR2), resolvin D4 (RvD4), resolvin D5 (RvD5), and resolvin E1 (RvE1), respectively, in mentioned groups. *P*-values were obtained by performing a Mann–Whitney *U* test. Only lipid mediators with *P* < 0.05 by Kruskal–Wallis test (Table 3) were represented.

The serum lipid metabolome was determined to measure differences in ω3-PUFA and lipid mediator (LM) profiles between the four groups: control group, mild, moderate, and severe COVID-19 patients. As shown in Fig. 2 and Table 3, prostaglandin E_2_ (PGE_2_) levels were significantly increased in all COVID-19 groups versus the control group (*P* < 0.05), and prostaglandin D_2_ (PGD_2_) was increased in the severe COVID-19 group versus less severe COVID-19 groups. No significant differences were found in other LMs from the AA cascade. Considering the docosahexaenoic acid (DHA) metabolome, the severe COVID-19 group showed significantly higher production of 14-HDHA than the control (*P* = 0.010) and mild groups (*P* = 0.021). Then, when looking at specific non-monohydroxylated SPMs generated from DHA, an increase in MaR2 was found in the severe COVID-19 group versus any other group. By contrast, a slight but statistically significant MaR2 reduction was found in the moderate COVID-19 versus the control group. Furthermore, significant increases in RvD5 levels were also found in severe and moderate COVID-19 groups versus controls and in RvD4 in mild COVID-19 versus control and moderate groups (*P* = 0.016 in both cases). Finally, concerning the eicosapentaenoic acid (EPA) metabolome, statistically significantly higher levels of RvE1 were found in severe COVID-19 versus all other groups (*P* < 0.05).

**Table 3:** Active lipid mediators and the precursor w-3 PUFA in controls and COVID-19 groups.

	Control (n = 7)	Mild (n = 8)	Moderate (n = 8)	Severe (n = 8)	*P-*value
EPA, ng/ml	143.9 (69.6–235.7)	94.1 (70.0–100.4)	65.0 (35.0–78.0)	90.8 (80.0–121.7)	0.146
DHA, ng/ml	74.0 (55.2–94.0)	41.9 (33.6–50.5)	48.2 (35.0–56.6)	44.5 (36.7–54.4)	0.053
DPA, ng/ml	139.7 (97.9–269.0)	144.0 (128.3–161.9)	132.1 (110.0–170.5)	169.3 (103.8–210.0)	0.801
18-HEPE, ng/ml	0.3 (0.3–0.8)	0.4 (0.3–0.4)	0.2 (0.2–0.5)	0.9 (0.6–1.4)	0.109
17-HDHA, ng/ml	1.6 (1.0–1.8)	2.2 (1.5–3.0)	1.6 (1.1–3.5)	4.2 (2.4–5.1)	0.135
14-HDHA, ng/ml	2.4 (1.9–4.1)	3.7 (2.4–8.8)	6.6 (3.1–9.3)	24.7 (5.9–44.0)	**0.021**
RvE1, pg/ml	0.0 (0.0–0.0)	0.0 (0.0–0.0)	0.0 (0.0–0.0)	112.6 (0.0–502.7)	**0.007**
RvD1, pg/ml	0.0 (0.0–0.0)	1.4 (0.0–5.5)	0.0 (0.0–3.9)	1.0 (0.0–2.6)	0.173
RvD2, pg/ml	4.4 (0.0–10.7)	9.1 (6.5–9.8)	9.1 (6.5–13.8)	5.1 (4.5–5.9)	0.362
RvD3, pg/ml	2.1 (0.8–3.2)	1.5 (0.5–2.1)	0.0 (0.0–0.9)	0.0 (0.0–0.5)	0.053
RvD4, pg/ml	0.0 (0.0–0.0)	0.5 (0.0–1.1)	0.0 (0.0–0.0)	0.0 (0.0–0.2)	**0.011**
RvD5, pg/ml	0.0 (0.0–0.0)	0.0 (0.0–11.5)	15.0 (7.6–30.1)	24.0 (11.6–35.6)	**0.021**
MaR1, pg/ml	59.5 (45.7–83.0)	36.7 (28.1–49.2)	40.9 (30.8–63.6)	64.0 (33.9–79.3)	0.416
MaR2, pg/ml	8.1 (5.1–9.3)	5.5 (3.4–7.7)	3.0 (1.9–5.9)	14.5 (13.5–20.5)	**0.007**
PD1, pg/ml	0.0 (0.0–0.0)	1.2 (0.0–2.1)	0.6 (0.0–1.9)	1.2 (0.0–1.9)	0.124
PDX, pg/ml	19.6 (15.0–28.3)	28.8 (12.5–33.6)	17.2 (4.9–25.3)	9.0 (6.8–44.0)	0.798
LXA_4_, pg/ml	4.9 (3.1–5.2)	3.5 (0.0–4.7)	3.1 (2.5–5.7)	4.3 (2.2–15.5)	0.803
LXB_4_, pg/ml	0.0 (0.0–7.5)	2.1 (0.0–4.8)	0.0 (0.0–5.5)	4.7 (0.0–74.1)	0.581
PGE_2_, pg/ml	103.6 (93.2–114.1)	279.7 (123.5–376.1)	182.8 (158.8–242.3)	226.6 (153.8–383.1)	**0.042**
PGD_2_, pg/ml	9.2 (0.0–17.7)	0.0 (0.0–5.8)	3.1 (0.0–6.9)	25.0 (11.7–36.2)	**0.030**
PGF_2α_, pg/ml	176.5 (110.5–225.3)	415.0 (159.1–699.7)	241.2 (136.4–329.0)	327.3 (183.4–734.6)	0.453
TXB_2_, ng/ml	8.0 (6.4–18.1)	30.4 (5.9–74.7)	30.4 (15.8–39.3)	10.2 (6.2–12.5)	0.325
LTB_4_, pg/ml	110.1 (35.7–177.7)	233.3 (194.6–476.1)	264.9 (131.7–374.9)	248.7 (187.8–848.0)	0.092

Data expressed as median (25th–75th percentile); Statistical analysis by Kruskal–Wallis test, *P* < 0.05 was considered statistically significant. When lipids were not detected in samples, an arbitrary value of 0.001 was used for statistical analysis^32^. Abbreviations: eicosapentaenoic acid (EPA), docosahexaenoic acid (DHA), docosapentaenoic acid (DPA), hydroxyeicosapentaenoic acid (HEPE), hydroxy docosahexaenoic acid (HDHA), resolvin (Rv), maresin (MaR), protectin (PD), lipoxin (LX), prostaglandin (PG), tromboxane (TX), and leukotriene (LT).

Significant values are in [bold].

**Figure 3:**
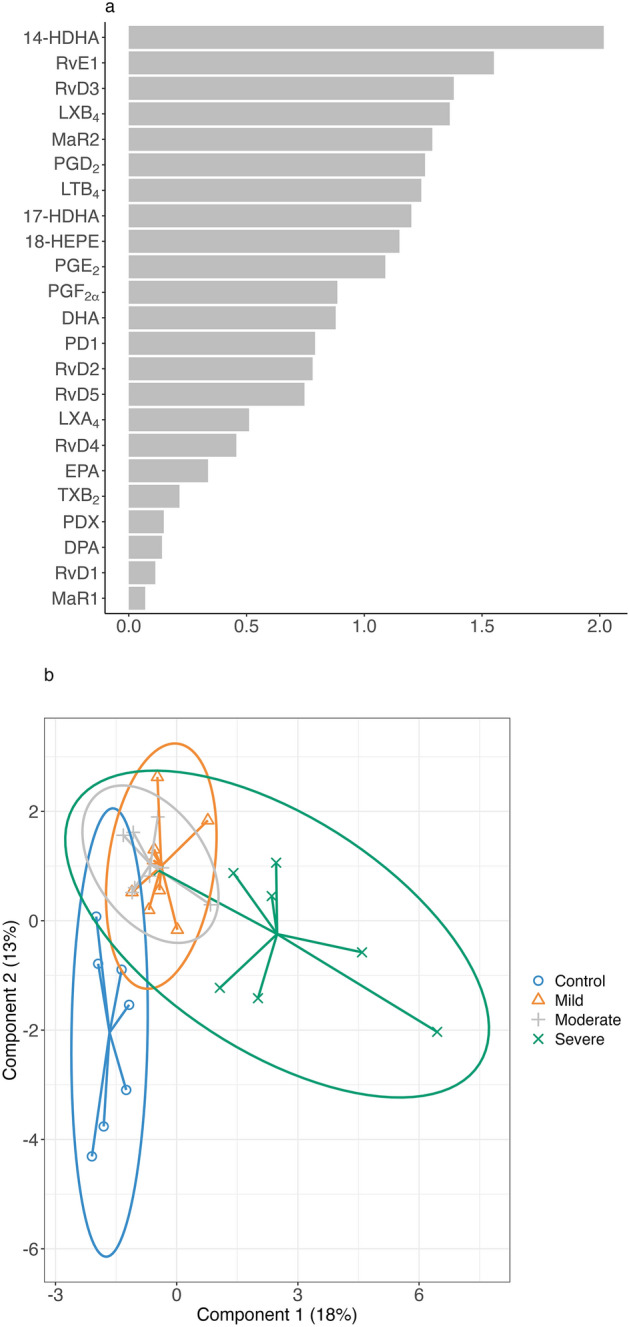
sPLS-DA analysis of active lipid mediators and precursor ω3-PUFA concentrations for discriminanting the four groups of patients (control, mild, moderate, and severe). (**a**) Variable importance in projection (VIP) scores of the lipid mediators and precursors. VIP larger than 1 highlights the most relevant indicators for identifying the four groups of study. Abbreviations: hydroxy docosahexaenoic acid (HDHA), resolvin (Rv), lipoxin (LX), maresin (MaR), prostaglandin (PG), leukotriene (LT), hydroxyeicosapentaenoic acid (HEPE), docosahexaenoic acid (DHA), protectin (PD), eicosapentaenoic acid (EPA), tromboxane (TX), and docosapentaenoic acid (DPA). **(b)** sPLS-DA sample plot with 95% confidence ellipse plots. A star plot displays arrows from each group centroid towards each individual sample.

Having obtained significant differences in the bivariate analysis between the groups, we conducted a sparse partial least squares discriminant analysis (sPLS-DA) based on concentrations of mediators to enable the selection of the most discriminative mediators to classify the groups. The sPLS-DA confirmed the contribution of 14-HDHA, RvE1, MaR2, PGD_2_, and PGE_2_ in the separation between each of the groups, with VIP values over 1. However, the VIP values for RvD4 and RvD5 mediators were lower than 0.75. Otherwise, RvD3, LXB_4_, LTB_4_, 17-hydroxy docosahexaenoic acid (17-HDHA), and 18-hydroxyeicosapentaenoic acid (18-HEPE) showed no significant difference between groups in the bivariate analysis but had a high discriminant capacity in the sPLS-DA analysis (Fig. 3a). The sPLS-DA sample plot automatically displays the group membership of each sample. In Fig. 3b, we can observe clear discrimination between the severe COVID group vs the others on the first component (x-axis) and in controls vs COVID-19 groups on the second component (y-axis).

## Discussion

This is a retrospective pilot study, with a reduced number of participants, conducted in order to get insight about the profiles of SPMs and other LMs in COVID-19 patients with different oxygen needs admitted to hospital, which remains largely unknown^18,29^.

Proper regulation of the inflammatory response is critical for successful pathogen elimination without affecting healthy tissues. Moreover, once the pathogen is cleared, inflammation must be resolved to avoid further tissue damage and damaged tissues must be repaired, for which pro-resolving lipid mediators, the SPMs, have emerged in recent years as key regulators. Most COVID-19 studies have focused on the role of inflammatory cytokines in COVID-19 disease^7,8,30,31^, with little attention paid to the mechanisms involved in the regulation of its activity and the resolution of inflammation. Our data suggest the presence of important amounts of bioactive lipids in serum from COVID-19 patients, with some of them especially upregulated in the most severe cases. Our findings are consistent with the report of increased LM concentrations in the bronchoalveolar lavage (BAL) fluid, tracheal aspirate (TA), and serum of COVID-19 patients^17,18,32^, showing that the lipid storm in these patients involves the mobilization of not only pro- but also anti-inflammatory lipids. These SPMs are generally considered to be produced after the class-switching phenomenon, appearing during the resolution phase of inflammation, but the mentioned studies have demonstrated their coexistence with pro-inflammatory lipid derivatives during the acute phase of inflammation or infection. In the same line, the induction of SPMs upon infection with viruses like influenza has been previously reported and has been proposed to act to limit their pathogenicity, but in some virulent strains, the upregulation may not be of sufficient magnitude to stop virus replication^33,34^. Something similar might be speculated in relation to severe COVID-19 where the upregulation of some SPMs, although with pro-resolving effects, could be an attempt to limit the infection/inflammation; this might be not enough to resolve severe disease, but a possible distortion in LM profiles due to the use of usual pharmacological compounds used for treatment COVID-19 cannot be excluded. In this line, some interactions have already been described, such as for the steroid dexamethasone that may act to limit pro-inflammatory eicosanoid generation or increase SPM production^35,36^. Other relevant interactions have also been described; for example, relative to the use of glucocorticoids as anti-inflammatory therapy during COVID-19; and also via the synthesis of lipid mediators, such as overexpression of the 2-arachidonoylglycerol or underexpression of the platelet-activating factor (PAF) induced by modification of lipid metabolism-enzyme gene expression^37^. Moreover, antiviral drugs, antibiotics, and immunosuppressants can induce liver injury^38^, which causes an inflammatory response that may influence LM profiles. For example, Kulkarni et al. described that lopinavir/ritonavir, the most widely used antiviral drug in our cohort, induced liver injury in 37.2% (remdesivir in 15.2%) of treated patients^39^. Thus, antiviral therapy could influence the LM profile in the severe group as it was more frequently used in this cohort. All COVID-19 patients showed an increase in the cyclooxygenase (COX) metabolite PGE_2_. Severe COVID-19 was characterized by 1) an increase of RvE1 and MaR2 levels versus non-affected donors and those in less severe COVID-19 groups; 2) an increase of 14-HDHA versus healthy donors and mild COVID-19 patients; 3) an increase of PGD_2_ versus less severe COVID-19 groups. Both severe and moderate COVID-19 groups showed increased RvD5 versus non-affected donors. Finally, moderate COVID-19 participants were characterized by a reduction in MaR2 compared to healthy donors.

It should be mentioned that at the study centre, serum was the most readily available tissue for extraction during the first wave of the pandemic. While SPMs are naturally produced during the coagulation process^40^, SPM levels are not comparable between plasma and serum. SPM levels are 10 to 100 times greater in serum than in human plasma, reflecting cellular activation processes and the contribution of SPMs not only to inflammation resolution but also to clot retraction and tissue repair^41^. Specifically, serum lipid mediator concentrations reflect the full capacity of circulating cells to produce lipid mediators upon activation, which alludes to the first-responders profile, rather than regular levels of these molecules in the circulation. As controls, serum from healthy donors obtained before the pandemic was used to avoid any contribution of asymptomatic or non-diagnosed cases with few symptoms in the data pool, ensuring that control samples were 100%-guaranteed COVID-free.

As mentioned above, concerning COX-derived metabolites, we observed an increase in PGE_2_ from AA in COVID-19 and it was identified by sPLS-DA analysis as one of the most relevant mediators to classify the groups. Although initially classified as a pro-inflammatory LM due to its positive correlation with the magnitude of the inflammatory stimulus, PGE_2_ is currently recognized for its pivotal role promoting the LM class switching, limiting the production of pro-inflammatory LMs and encouraging the start of SPM production^42,43^. During the inflammatory process, after PGE_2_ reaches its peak, production of the pro-resolving LXA_4_ from AA begins with the activation of lipoxygenase (LOX) pathways. A potential mechanism in which the failure to reach the PGE_2_ summit in COVID-19 might produce deficient LXA_4_ synthesis and lead to the failure of inflammation resolution has been previously suggested^43^. Nevertheless, the present study did not find altered levels of LXA_4_ during COVID-19 in comparison with healthy donors. Although no significant differences in PGE_2_ levels were found between the severe disease group and the other COVID-19 groups, additional studies including samples along the follow-up of the patient’s disease would be required to discard the role of PGE_2_ in disease severity. Since we do not know when PGE_2_ reaches its peak during COVID-19, we cannot conclude whether the absence of differences between mild and severe cases is affected by the sampling time in COVID-19 infection. Our results concerning the increase in PGE_2_ are in agreement with those found in influenza A virus (IAV) infection, where the increased levels of PGE_2_ cause inhibition of macrophage recruitment to the lungs, type I interferon release, and macrophagic apoptosis, enhancing the opportunities for IAV replication. In addition, PGE_2_ suppression acting through PGE-synthase 1 or the inhibition of EP2 and EP4 PGE_2_ receptors improves survival against lethal IAV infection and that is reversed by PGE_2_ administration^44^. Since all COVID-19 patients showed increased levels of PGE_2_ and it has been previously reported that PGE_2_ triggers platelet aggregation via EP3 receptor activation, increasing the risk of thrombosis, it is tempting to speculate that increased levels of PGE_2_ in COVID-19 might be related to coagulation disorders, a common complication found in COVID-19 patients^45–48^. Although we did not classify patients according to the severity of coagulation disorders but concerning pulmonary involvement, those requiring the hardest interventions for oxygen administration, such as intubation and mechanical ventilation, showed not only similar high levels of PGE_2_ but reduced levels of aPTT when compared to those with less severe COVID-19. Abnormalities in coagulation parameters suggesting hypercoagulability, such as increased D-dimer, mild thrombocytopenia, and prolonged prothrombin time (PT), have been associated with increased risk of death. These have been previously reported in COVID-19^38,49–51^ but contradictory results were found for aPTT, probably due to the influence of other factors such as C-reactive protein, fibrinogen, and factor VIII, or the presence of heparin or lupus anticoagulants in aPTT measurements^52^. In line with our results, increased levels of PGE_2_ were also found in BAL fluid from severe COVID-19 patients^18^ and in serum from COVID-19 patients^32^ compared to healthy controls. However, contradictory results have been found concerning the association of PGE_2_ with COVID-19 severity. Schwarz et al.’s study (n = 18/20 patients per group) showed that serum levels are negatively associated with the severity, whereas a positive correlation was described by Ricke-Hoch et al.^29,53^. We did not find differences in PGE_2_ levels between COVID-19 patients when classifying them according to lung damage severity. Differences among these studies could be due to variations in the criteria used for allocating patients to groups with different severity. By contrast, the present study did not find differences in another metabolite, TXB_2_, associated in general with bronchoconstriction and coagulatory properties implied in platelet and inflammatory cell activation. Our findings are in line with those reported in plasma in critical COVID-19 patients^17^, but are not in agreement with those found in BAL fluid or TA^17,18,54^ or in serum^32^. Discrepancies between the mentioned studies may be related to differences in study designs and methodology, such as the type of sample matrix, the method sensitivity, sample processing, or in the definition of the control group. Thus, studies with larger sample sizes and sharing the same criterion for classifying patients according to disease severity groups are needed to clarify this point. Unfortunately, we cannot ensure hemostasis dysregulation in our cohort due to the lack of data relative to the coagulation profile in the control group, but interestingly, no significant differences were found in PT or platelets count among COVID-19 groups.

PGD_2_ is an important prostaglandin for respiratory viruses and is more highly expressed than PGE_2_ in BAL fluid from healthy subjects^55,56^. It usually goes up with ageing and is also stimulated by SARS-CoV RNA via upregulation of COX enzymes^57^. PGD_2_ can display both pro- and anti-inflammatory effects via activation of DP1 and DP2 receptors. Current knowledge on PGD_2_ is not sufficient to assert its positive or negative role in COVID-19. Whereas some authors have proposed the use of DP1 receptor antagonists to raise respiratory dendritic cell migration, T cell responses, and virus clearance in lungs to protect against severe disease manifestations by using animal models infected with SARS-CoV-1 and murine-adapted SARS-CoV-2^56,58^, others have claimed the beneficial effects of PGD_2_/DP1 signalling in the prevention of inflammasome hyperactivation in the brain of animal models infected with neurotropic coronavirus^59^ or in alleviating inflammation and vascular permeability, proposing conversely the preservation of the PGD_2_/DP1 axis and the blockage of PGD_2_/DP2 signalling^60^.

Our detailed lipidomic analysis provides evidence of altered bioactive metabolite levels arising from the LOX pathways too, which might reflect the response to attempt to reduce the exacerbated inflammatory response especially in the most severe cases.

The DHA metabolome was of particular interest. Significantly increased 14-HDHA levels were found in the severe COVID-19 versus control or mild groups, as previously reported by Archambault et al. in BAL fluid in severe COVID-19 versus controls^18^. 14-HDHA also showed the highest VIP score in the sPLS-DA analysis. Likewise, although we found no alteration in serum 17-HDHA levels, contrary to that previously reported in BAL fluid and serum^18,32^, similar to Archambault et al., increases in RvD5 concentrations, a 17-HDHA downstream bioactive lipid, were found in the severe COVID-19 versus control in both studies. In addition, the present work found increased levels of RvD5 when comparing moderate and severe COVID-19 groups versus controls. In addition, a significant increase in serum levels of MaR2 in COVID-19 patients with mechanical ventilation was found when compared to control, mild, and moderate COVID-19 groups, and a small but significant reduction in MaR2 levels in moderate COVID-19 versus the control group. Moreover, the sPLS-DA identified MaR2 as a very important mediator in groups’ discrimination. These results suggest that MaR2 might be a potential biomarker of poor prognosis. Maresins are anti-inflammatory and pro-resolving SPMs produced by macrophages from 14-HpDHA by 12/15 LOX enzymes^61^, linked with a potent blockade of neutrophil infiltration, but MaR2 seems to be less powerful than MaR1 to enhance the human macrophage phagocytosis of apoptotic polymorphonuclear cells^62^. Higher values of MaR2 have been previously described in COVID-19 patients^32^, whereas Palmas et al. reported elevation of MaR1^63^ relative to healthy controls.

Differences found between these studies could be due to variations in patients’ allocation, in the time from disease onset to sample extraction that could also be influenced by the type of study, retrospective or prospective; the reduced sample size of most studies, and the methodology for sample extraction and post-extraction processing. In addition, variations in SPM levels in the different biofluids, such as distinct blood fractions or BAL fluid, could contribute to the differences observed too as previously reported for PGE_2_, TXB_2_, and 6-Trans-LTB_4_, that increased in TA samples, but not in the plasma of COVID-19 patients^17^. In our opinion, a joint effort should be made by all researchers to establish a consensus on the way to classify patients and an appropriate sampling time, so that the comparison of the results could increase our understanding of the clinical differences found in patients infected with the SARS-CoV-2.

Apart from SPM analysis, clinical parameters showed increased BUN in moderate to severe versus mild COVID-19 groups, which is in line with results previously published which pointed out the role of BUN as an independent factor associated with a high risk of oxygen requirements and one of the three prognostic factors, together with age and body mass index, included in a risk nomogram for oxygen requirement^64^.

In conclusion, the potent inflammatory response to SARS-CoV-2 infection not only involves pro-inflammatory cytokines but also triggers the release of anti-inflammatory LMs that can be quantified from easily accessible peripheral blood samples subjected to the blood-clotting process. We have found a relevant increase in MaR2 in severe COVID-19 disease versus not only healthy donors but also versus all other COVID-19 groups, which could help clinicians to distinguish moderate from severe patients. On the other hand, although increased RvD5 levels were found in moderate to severe groups versus non-affected individuals, it was not possible to identify patients with a mild course of the disease by SPM analysis. These findings, together with those previously reported, will motivate future research into LMs and resolution pathways that could lead to the discovery of new therapeutic targets against SARS-CoV-2 infection.

Our study has some limitations. Firstly, this is a pilot study, with reduced sample size, studying the SPM metabolome in three different groups of COVID-19 severity based on lung involvement. Further studies with larger sample sizes should be performed to validate the present results. We cannot ensure that the results found are COVID-19-specific because we did not have the possibility to include a control group of patients with pulmonary disease of comparable severity but not attributable to SARS-CoV-2. It should be mentioned that regardless of the control group**’**s lower age versus moderate and severe COVID groups, results in healthy controls show similar or lower concentrations of SPMs whereas, in general, it is accepted that ageing is linked with chronic low-grade inflammation which might lead to a lower pro-resolving capacity^65,66^. Therefore, it can be ensured that those SPMs that were found to be upregulated in COVID-19 groups were really high but it might be possible that age differences masked alterations in other SPMs that showed similar concentrations to those observed in the control group. However**,** it has been reported that age does not alter the ability to develop a pro-resolution reaction^32^. Although there were no statistically significant differences between groups concerning comorbidities, the variety of drugs that these patients receive as habitual therapies or differences found in the severe COVID-19 group concerning the antiviral treatment could influence LM generation. Finally, although an increase in serum PGE_2_ level on infection was found in our study, we measured PGE_2_ only at a single specific moment during the disease. Sequential measurements of this metabolite during the whole process would be useful to determine whether PGE_2_ keeps increasing or not, in the most severe cases. Finally, samples were collected during the first wave of the pandemic and, due to the health system overload at the time, some clinical data such as D-dimers were not registered.

## Materials and methods

### Patients and ethics statement

This study includes samples from 24 hospitalized COVID-19 patients (see Tables 1 and 2 for demographics and clinical information of study participants) collected in the period from March 2020 to July 2020. According to the severity criteria at the time of hospital admission, patients were classified into three groups depending on oxygen therapy needs: mild cases (n = 8) that do not need oxygen therapy, moderate cases (n = 8) needing flow oxygen therapy, and severe cases (n = 8) with severe ARDS that requires intubation and mechanical ventilation in ICUs. An additional control group of non-infected subjects collected among voluntary blood donors recruited in the Aragon Blood and Tissue Bank before the pandemic, throughout the year 2018 and the beginning of 2019, was used. This study was approved by the Ethical Committee of Clinical Research of Aragon (CEICA), project number PI20/165. All participants signed the informed consent form, and the study was conducted in accordance with the principles stated in the Declaration of Helsinki (Ethical Principles for Medical Research Involving Human Subjects, Helsinki, Finland, 1964) and as amended in Fortaleza, Brazil in 2013.

### Sample collection and processing

Serum samples used for LM measurements were provided by the Biobank of the Aragon Health System, part of the Spanish National Biobanks Network, and processed following standard operating procedures with the appropriate approval of the Ethics and Scientific Committees. In brief, peripheral blood samples were collected using BD Vacutainer^®^ SST™ tubes. The mean time from the onset of COVID-19 symptoms to blood withdrawal was 7 days. The sample tubes were left in an upright position for 30 min at room temperature for complete clot formation and then were centrifuged at 1500×*g* for 10 min at room temperature. Serum samples were conserved at 4 °C overnight and excess serum from diagnosis was then provided for research and frozen at − 80 °C. This serum was the only biological sample available during the beginning of the pandemic. Other determinations in blood were made using the standardized methods at the hospital and data related to these clinical parameters were extracted from the patients’ clinical history.

### ***Lipid mediator extraction and profiling (LC–MS/MS)***

To characterize the effect of coronavirus on the production of pro-inflammatory and pro-resolving LMs, the serum concentrations of 23 variables were determined by LC–MS/MS: ω-3 PUFAs (eicosapentaenoic acid, EPA; docosahexaenoic acid, DHA; docosapentaenoic acid, DPA), their monohydroxylated LMs (18-HEPE, 17-HDHA, and 14-HDHA), pro-inflammatory arachidonic acid (AA) derivatives (prostaglandins: PGE_2_, PGD_2_, PGF_2α_; thromboxane B_2_, TXB_2_; and leukotriene B_4_, LTB_4_), and SPMs including resolvins from EPA and DHA (RvE1, RvD1, RvD2, RvD3, RvD4, and RvD5), maresins and protectins from DHA (MaR1, MaR2, PD1, and PDX), and lipoxins from AA (LXA_4_ and LXB_4_).

LMs were extracted from human serum samples according to the following solid-phase extraction (SPE) method. Each sample (serum, 1 ml) stored at − 80 °C was thawed on ice. Internal labelled standards d8-5-HETE, d5-RvD2, d5-LXA_4_, d4-LTB_4_, and d4-PGE_2_ (500 pg each, Cayman Chemical Company) in 4 ml of methanol (Methanol Optima LC/MS Grade, Fisher Chemical) were added to each sample. Known concentrations of LMs in labelled standards were used for quantification purposes and posterior calculations on the recovery of LMs. Calibration curves were obtained using synthetic and authentic LM mixtures, including d4-LTB_4_, d5-LXA_4_, d4-PGE_2_, d5-RvD2, 5(S)-HETE-d8, RvD1, RvD2, RvD3, RvD4, RvD5, PD1, PDX, MaR1, MaR2, RvE1, LXA_4_, LXB_4_, PGE_2_, PGD_2_, PGF_2α_, TXB_2_, and LTB_4_ at 1, 5, 25, 50, 100, and 200 pg. Linear calibration curves for each compound were obtained with R^2^ values between 0.993 and 0.999. Then, the samples were placed at − 80 °C for 30 min for protein precipitation. Next, the probes were centrifuged (2000×*g*, 10 min, 4 °C). The supernatant was obtained from each sample. SPE was performed according to optimized and reported methods^41,67^. Furthermore, samples were quickly acidified to pH = 3.5 with 9 ml of acidic water (HCl) just prior to loading onto SPE columns (100 mg, 10 ml, Biotage) and pH neutralization with 4 ml of Milli-Q water, followed by a washing step with 4 ml of n-hexane. After that, compounds were eluted with 9 ml of methyl formate. Extracts from the SPE were dried under a gentle stream of nitrogen and immediately after were resuspended in methanol/water (50 : 50 vol/vol) (MeOH/Water Optima LC/MS Grade, Fisher Chemical, both) before injection into an LC–MS/MS system.

### Targeted LC–MS/MS acquisition parameters

The LC–MS/MS system consisted of a Qtrap 5500 (Sciex) equipped with a Shimadzu LC-20AD HPLC. A Kinetex Core–Shell LC-18 column (100 mm × 4.6 mm × 2.6 μm, Phenomenex) was housed in a column oven maintained at 50 °C. A binary eluent system of LC–MS/MS-grade water (A) (Fisher Chemical) and LC–MS/MS-grade methanol (Fisher Chemical) (B), both with 0.01% (v/v) of acetic acid, were used as the mobile phase. LMs were eluted in a gradient programme with respect to the composition of B as follows: 0.0–2.0 min, 50%; 2.0–14.5 min, 80%; 14.6–25.0 min; 98%. The flow rate was 0.5 ml/min.

The QTRAP 5500 was operated in negative ionization mode, using scheduled Multiple Reaction Monitoring (MRM) coupled with information-dependent acquisition (IDA) and an Enhanced Product Ion (EPI) scan. Each LM parameter (collision energy, target retention time, and specific first and third quadrupole mass transitions) were optimized according to reported methods^67,68^. For monitoring and quantification purposes, the amounts of LMs of interest were estimated as the area under the peak, specifically using MRM with MS/MS matching signature ion fragments for each molecule (at least six diagnostic ions; < 0.1 pg was considered below the limit of detection) using published criteria^68^. The lower limits of quantification (LLOQ) were determined by analysing serial dilutions of the lower calibrator as the concentrations of each analyte with a signal/noise ≥ fivefold the signal/noise of a blank solution, according to the guidelines of the US Food and Drug administration^69^, and are shown in Table 4. The laboratory analyses were performed by Solutex GC, SL.

**Table 4:** Lower limits of quantification.

	LLOQ (pg/ml)
DHA	0.02
DPA	0.15
EPA	0.01
18-HEPE	0.24
17-HDHA	0.33
14-HDHA	0.08
RvE1	0.21
RvD1	0.64
RvD2	0.97
RvD3	0.30
RvD4	0.42
RvD5	0.25
MaR1	0.74
MaR2	0.18
PD1	0.15
PDX	0.30
LXA_4_	0.28
LXB_4_	0.25
PGE_2_	0.30
PGD_2_	0.17
PGF_2α_	0.43
TXB_2_	0.29
LTB_4_	0.25

The LLOQ was defined as the analyte concentration with a signal/noise ≥ fivefold the signal/noise of the blank solution. Abbreviations: docosahexaenoic acid (DHA), docosapentaenoic acid (DPA), eicosapentaenoic acid (EPA), hydroxyeicosapentaenoic acid (HEPE), hydroxy docosahexaenoic acid (HDHA), resolvin (Rv), maresin (MaR), protectin (PD), lipoxin (LX), prostaglandin (PG), tromboxane (TX), and leukotriene (LT).

### Statistical analysis

An initial descriptive exploratory analysis of all clinical variables was carried out. Continuous variables were expressed as the median with interquartile range (25–75th percentile), whereas qualitative variables were expressed as frequencies and percentages. Differences between population groups were evaluated with the Fisher test for qualitative variables and with the Mann–Whitney test or Kruskal–Wallis test for continuous variables. For statistical analysis, non-detectable results were given an arbitrary value of 0.001^32^. Normality was tested using the Shapiro–Wilk test.

Spared partial least squares discriminant analysis (sPLS-DA) was applied to identify LMs that discriminate the four groups of patients (control, mild, moderate, and severe). Variable importance in projection (VIP) allowed their classification according to their explanatory power of the variable ‘group of the study’; predictors with a large VIP were the most relevant.

The level of bilateral significance in the study was established at 0.05. Statistical analysis was performed using the R v.3.5.3 programming language (The R Foundation for statistical computing, Vienna, Austria). In particular, the mixOmics R package was used to compute the sPLS-DA^70^.

## Data Availability

The data supporting the present study are available in the article or will be obtained from the corresponding author upon request.

## References

[1] Huang C (2020). Clinical features of patients infected with 2019 novel coronavirus in Wuhan, China. Lancet.

[2] Tian W (2020). Predictors of mortality in hospitalized COVID-19 patients: A systematic review and meta-analysis. J. Med. Virol..

[3] Salık F (2021). Liver function as a predictor of mortality in COVID-19: A retrospective study. Ann. Hepatol..

[4] España PP (2021). Predictors of mortality of COVID-19 in the general population and nursing homes. Intern. Emerg. Med..

[5] Tavares LP, Teixeira MM, Garcia CC (2017). The inflammatory response triggered by Influenza virus: A two edged sword. Inflamm. Res..

[6] Chen L (2017). Inflammatory responses and inflammation-associated diseases in organs. Oncotarget.

[7] Ragab D, Salah Eldin H, Taeimah M, Khattab R, Salem R (2020). The COVID-19 cytokine storm; What we know so far. Front. Immunol..

[8] Wang J, Jiang M, Chen X, Montaner LJ (2020). Cytokine storm and leukocyte changes in mild versus severe SARS-CoV-2 infection: Review of 3939 COVID-19 patients in China and emerging pathogenesis and therapy concepts. J. Leukoc. Biol..

[9] Luo XH, Zhu Y, Mao J, Du RC (2021). T cell immunobiology and cytokine storm of COVID-19. Scand. J. Immunol..

[10] da Silva-Neto PV (2022). Matrix metalloproteinases on severe COVID-19 lung disease pathogenesis: cooperative actions of MMP-8/MMP-2 axis on immune response through HLA-G shedding and oxidative stress. Biomolecules.

[11] Sorokin AV (2020). COVID-19-Associated dyslipidemia: Implications for mechanism of impaired resolution and novel therapeutic approaches. FASEB J..

[12] D’Ardes D (2021). Metabolic changes in SARS-CoV-2 infection: Clinical data and molecular hypothesis to explain alterations of lipid profile and thyroid function observed in COVID-19 patients. Life (Basel Switz.).

[13] Rezaei A, Neshat S, Heshmat-Ghahdarijani K (2022). Alterations of lipid profile in COVID-19: A narrative review. Curr. Probl. Cardiol..

[14] Headland SE, Norling LV (2015). The resolution of inflammation: Principles and challenges. Semin. Immunol..

[15] Saito P (2018). The lipid mediator resolvin D1 reduces the skin inflammation and oxidative stress induced by UV irradiation in hairless mice. Front. Pharmacol..

[16] Li T (2022). Maresin 1 improves cognitive decline and ameliorates inflammation and blood-brain barrier damage in rats with chronic cerebral hypoperfusion. Brain Res..

[17] Pérez MM (2022). Acetylcholine, fatty acids, and lipid mediators are linked to COVID-19 severity. J. Immunol..

[18] Archambault AS (2021). Lipid storm within the lungs of severe COVID-19 patients: Extensive levels of cyclooxygenase and lipoxygenase-derived inflammatory metabolites. FASEB J.

[19] Serhan CN, Levy BD (2018). Resolvins in inflammation: Emergence of the pro-resolving superfamily of mediators. J. Clin. Invest..

[20] Barker G (2021). Lipid and lipoprotein dysregulation in sepsis: Clinical and mechanistic insights into chronic critical illness. J. Clin. Med..

[21] Pontes-Arruda A, Aragão AMA, Albuquerque JD (2006). Effects of enteral feeding with eicosapentaenoic acid, γ-linolenic acid, and antioxidants in mechanically ventilated patients with severe sepsis and septic shock*. Crit. Care Med..

[22] Pontes-Arruda A, DeMichele S, Seth A, Singer P (2008). The use of an inflammation-modulating diet in patients with acute lung injury or acute respiratory distress syndrome: A meta-analysis of outcome data. J. Parenter. Enter. Nutr..

[23] Hosny M, Nahas R, Ali S, Elshafei SA, Khaled H (2013). Impact of oral omega-3 fatty acids supplementation in early sepsis on clinical outcome and immunomodulation. Egypt. J. Crit. Care Med..

[24] Langlois PL, D’Aragon F, Hardy G, Manzanares W (2019). Omega-3 polyunsaturated fatty acids in critically ill patients with acute respiratory distress syndrome: A systematic review and meta-analysis. Nutrition.

[25] Mancuso P (1997). Effects of eicosapentaenoic and γ-linolenic acid on lung permeability and alveolar macrophage eicosanoid synthesis in endotoxic rats. Crit. Care Med..

[26] Mancuso P (1997). Dietary fish oil and fish and borage oil suppress intrapulmonary proinflammatory eicosanoid biosynthesis and attenuate pulmonary neutrophil accumulation in endotoxic rats. Crit. Care Med..

[27] Bistrian BR (2020). Parenteral fish-oil emulsions in critically Ill COVID-19 emulsions. J. Parenter. Enter. Nutr..

[28] Torrinhas RS, Calder PC, Waitzberg DL (2020). Response to Bistrian BR. Parenteral fish-oil emulsions in critically Ill COVID-19 emulsions. J. Parenter. Enter. Nutr..

[29] Schwarz B (2021). Cutting edge: Severe SARS-CoV-2 infection in humans is defined by a shift in the serum lipidome, resulting in dysregulation of eicosanoid immune mediators. J. Immunol..

[30] Hu B, Huang S, Yin L (2021). The cytokine storm and COVID-19. J. Med. Virol..

[31] Quirch M, Lee J, Rehman S (2020). Hazards of the cytokine storm and cytokine-targeted therapy in patients with COVID-19: Review. J. Med. Internet Res..

[32] Turnbull J (2022). Serum levels of proinflammatory lipid mediators and specialized proresolving molecules are increased in patients with severe acute respiratory syndrome coronavirus 2 and correlate with markers of the adaptive immune response. J. Infect. Dis..

[33] Morita M (2013). The lipid mediator protectin D1 inhibits influenza virus replication and improves severe influenza. Cell.

[34] Tam VC (2013). Lipidomic profiling of influenza infection identifies mediators that induce and resolve inflammation. Cell.

[35] Pyrillou K, Chairakaki AD, Tamvakopoulos C, Andreakos E (2018). Dexamethasone induces ω3-derived immunoresolvents driving resolution of allergic airway inflammation. J. Allergy Clin. Immunol..

[36] Koenis DS (2021). Disrupted resolution mechanisms favor altered phagocyte responses in COVID-19. Circ. Res..

[37] de Carvalho JCS (2023). The interplay among glucocorticoid therapy, platelet-activating factor and endocannabinoid release influences the inflammatory response to COVID-19. Viruses.

[38] D’Ardes D (2022). Impaired coagulation, liver dysfunction and COVID-19: Discovering an intriguing relationship. World J. Gastroenterol..

[39] Kulkarni AV (2020). Systematic review with meta-analysis: Liver manifestations and outcomes in COVID-19. Aliment. Pharmacol. Ther..

[40] Norris PC, Libreros S, Chiang N, Serhan CN (2017). A cluster of immunoresolvents links coagulation to innate host defense in human blood. Sci. Signal..

[41] Colas RA, Shinohara M, Dalli J, Chiang N, Serhan CN (2014). Identification and signature profiles for pro-resolving and inflammatory lipid mediators in human tissue. Am. J. Physiol. Cell Physiol..

[42] Holopainen M (2019). Polyunsaturated fatty acids modify the extracellular vesicle membranes and increase the production of proresolving lipid mediators of human mesenchymal stromal cells. Biochim. Biophys. Acta Mol. Cell Biol. Lipids.

[43] Das UN (2020). Bioactive lipids as mediators of the beneficial action(s) of mesenchymal stem cells in COVID-19. Aging Dis..

[44] Coulombe F (2014). Targeted prostaglandin E2 inhibition enhances antiviral immunity through induction of type I interferon and apoptosis in macrophages. Immunity.

[45] Gross S, Tilly P, Hentsch D, Vonesch JL, Fabre JE (2007). Vascular wall-produced prostaglandin E2 exacerbates arterial thrombosis and atherothrombosis through platelet EP3 receptors. J. Exp. Med..

[46] Bikdeli B (2020). COVID-19 and thrombotic or thromboembolic disease: Implications for prevention, antithrombotic therapy, and follow-up: JACC state-of-the-art review. J. Am. Coll. Cardiol..

[47] Zaid Y (2020). Platelets can associate with SARS-CoV-2 RNA and are hyperactivated in COVID-19. Circ. Res..

[48] Hottz ED (2020). Platelet activation and platelet-monocyte aggregate formation trigger tissue factor expression in patients with severe COVID-19. Blood.

[49] Levi M, Thachil J, Iba T, Levy JH (2020). Coagulation abnormalities and thrombosis in patients with COVID-19. Lancet. Haematol..

[50] Letícia de Oliveira Toledo S, Sousa Nogueira L, das Graças Carvalho M, Romana Alves Rios D, de Barros Pinheiro M (2020). COVID-19: Review and hematologic impact. Clin. Chim. Acta..

[51] Rahman A (2021). Hematological abnormalities in COVID-19: A narrative review. Am. J. Trop. Med. Hyg..

[52] Devrees K (2021). COVID-19-related laboratory coagulation findings. Int. J. Lab. Hematol..

[53] Ricke-Hoch M (2021). Impaired immune response mediated by prostaglandin E2 promotes severe COVID-19 disease. PLoS ONE.

[54] Zaid Y (2021). Chemokines and eicosanoids fuel the hyperinflammation within the lungs of patients with severe COVID-19. J. Allergy Clin. Immunol..

[55] Ripon MAR, Bhowmik DR, Amin MT, Hossain MS (2021). Role of arachidonic cascade in COVID-19 infection: A review. Prostaglandins Other Lipid Mediat..

[56] Robb CT, Goepp M, Rossi AG, Yao C (2020). Non-steroidal anti-inflammatory drugs, prostaglandins, and COVID-19. Br. J. Pharmacol..

[57] Rizk JG (2020). Pharmaco-immunomodulatory therapy in COVID-19. Drugs.

[58] Roy Wong, L. Y. *et al.* Eicosanoid signaling as a therapeutic target in middle-aged mice with severe COVID-19. *bioRxiv Prepr. Serv. Biol.* (2021) 10.1101/2021.04.20.440676.

[59] Vijay R (2017). Virus-induced inflammasome activation is suppressed by prostaglandin D 2/DP1 signaling. Proc. Natl. Acad. Sci. U. S. A..

[60] Gupta A, Chander Chiang K (2020). Prostaglandin D 2 as a mediator of lymphopenia and a therapeutic target in COVID-19 disease. Med. Hypotheses.

[61] Serhan CN (2009). Maresins: novel macrophage mediators with potent antiinflammatory and proresolving actions. J. Exp. Med..

[62] Deng B (2014). Maresin biosynthesis and identification of maresin 2, a new anti-inflammatory and pro-resolving mediator from human macrophages. PLoS ONE.

[63] Palmas F (2021). Dysregulated plasma lipid mediator profiles in critically ill COVID-19 patients. PLoS ONE.

[64] Goto N (2021). The usefulness of a combination of age, body mass index, and blood urea nitrogen as prognostic factors in predicting oxygen requirements in patients with coronavirus disease 2019. J. Infect. Chemother..

[65] Calder PC (2017). Health relevance of the modification of low grade inflammation in ageing (inflammageing) and the role of nutrition. Ageing Res. Rev..

[66] Calder PC (2020). Eicosapentaenoic and docosahexaenoic acid derived specialised pro-resolving mediators: Concentrations in humans and the effects of age, sex, disease and increased omega-3 fatty acid intake. Biochimie.

[67] English JT, Norris PC, Hodges RR, Dartt DA, Serhan CN (2017). Identification and profiling of specialized pro-resolving mediators in human tears by lipid mediator metabolomics. Prostaglandins. Leukot. Essent. Fatty Acids.

[68] Dalli J, Colas RA, Walker ME, Serhan CN (2018). Lipid mediator metabolomics via LC-MS/MS profiling and analysis. Methods Mol. Biol..

[69] FDA & CDER. Bioanalytical Method Validation. Guidance for Industry. U.S. Department of Health and Human Services. (2018).

[70] Rohart F, Gautier B, Singh A, Lê Cao KA (2017). mixOmics: An R package for ’omics feature selection and multiple data integration. PLoS Comput. Biol..

